# Long-term impact of late pulmonary hypertension requiring medication in extremely preterm infants with severe bronchopulmonary dysplasia

**DOI:** 10.1038/s41598-024-58977-w

**Published:** 2024-04-15

**Authors:** Chan Kim, Sumin Kim, Hanna Kim, Jieun Hwang, Seung Hyun Kim, Misun Yang, So Yoon Ahn, Se In Sung, Yun Sil Chang

**Affiliations:** 1 Department of Pediatrics, Samsung Medical Center, Sungkyunkwan University School of Medicine, 81 Irwon-ro, Gangnam-ku, Seoul, 06351 Republic of Korea; 2 Department of Clinical Research Design and Evaluation, Samsung Advanced Institute for Health Sciences & Technology (SAIHST), Sungkyunkwan University, Seoul, Republic of Korea; 3 Cell and Gene Therapy Institute for Future Medicine, Samsung Medical Center, Seoul, Republic of Korea; 4 Department of Health Science and Technology, Samsung Advanced Institute for Health Sciences & Technology (SAIHST), Sungkyunkwan University, Seoul, Republic of Korea

**Keywords:** Hypertension, Neonatology, Preterm birth, Neurodevelopmental disorders

## Abstract

This study investigated whether late pulmonary hypertension (LPH) independently increases the risk of long-term mortality or neurodevelopmental delay (NDD) in extremely preterm infants (EPIs) with severe bronchopulmonary dysplasia (BPD). Using prospectively collected data from the Korean Neonatal Network, we included EPIs with severe BPD born at 22–27 weeks’ gestation between 2013 and 2021. EPIs having severe BPD with LPH (LPH, n = 124) were matched 1:3 with those without pulmonary hypertension (PH) as controls (CON, n = 372), via propensity score matching. LPH was defined as PH with the initiation of medication after 36 weeks’ corrected age (CA). Long-term mortality after 36 weeks’ CA or NDD at 18–24 months’ CA was analyzed. NDD was assessed using composite scores based on various neurodevelopmental assessment modalities. LPH had significantly higher long-term mortality or NDD (45.2% vs. 23.1%, *P* < 0.001), mortality (24.2% vs. 4.84%, *P* < 0.001), and NDD (68.4% vs. 37.8%, *P* = 0.001), respectively than CON, even after adjusting for different demographic factors. Multivariable regression demonstrated that LPH independently increased the risk of mortality or NDD (adjusted odds ratio, 1.95; 95% confidence intervals, 1.17–3.25). When LPH occurs in EPIs with severe BPD, special monitoring and meticulous care for long-term survival and neurodevelopment are continuously needed.

## Introduction

The survival rates of extremely preterm infants (EPIs) have been improved owing to the advances in neonatal intensive care; however, this improvement is accompanied by a concerning rise in bronchopulmonary dysplasia (BPD) prevalence among this population^1,2^. BPD not only leads to increased mortality and respiratory morbidity but also contributes to adverse long-term neurodevelopmental outcomes^3–6^. Notably, the negative impact on these outcomes becomes more pronounced as BPD severity worsens, making severe BPD a significant healthcare concern for affected EPIs even after discharge from the neonatal intensive care unit (NICU)^3–6^.

BPD-associated late pulmonary hypertension (PH) is a cardiorespiratory complication of BPD, typically evident after a corrected age (CA) of 36 weeks^7–9^. Late PH emerges as a significant challenge for EPIs with severe BPD owing to its strong correlation with BPD severity^10,11^. Despite the varied reported incidence owing to different screening protocols and diagnostic criteria across institutions^7,8^, infants with BPD-associated late PH consistently face significantly higher mortality rates than those with BPD alone^7–11^. The high mortality rate makes investigations on the long-term outcomes of infants with late PH challenging. Additionally, the heterogeneity among institutions hinders the feasibility of multi-center studies to investigate long-term outcomes. Sporadic studies about them have been reported. One retrospective multi-center study shows significantly increased re-hospitalization rates up to 1 year following NICU discharge among very preterm infants with severe BPD and late PH compared with those with severe BPD alone^12^. Additionally, only two single-center studies have indicated that EPIs with BPD experience worse long-term growth and neurodevelopment when exposed to late PH^13,14^. Therefore, investigating the long-term impact of late PH on EPIs with severe BPD is essential as this population remains at high risk of mortality and poor neurodevelopment even after NICU discharge. This study aimed to assess whether late PH independently increases the risk of long-term mortality or neurodevelopmental delay (NDD) at a CA of 18–24 months in EPIs with severe BPD using a large national prospective cohort, the Korean Neonatal Network (KNN).

## Methods

### Data source

This cohort study used a de-identified dataset from the KNN and was approved by the Committee of Ethics and Publication of the KNN. The KNN is a national cohort registry launched in 2013, supported by the Korea National Institute of Health (KNIH), and prospectively collects data on very low birth weight infants (VLBWIs) admitted to > 70 participating NICUs covering > 80% of VLBWIs born in Korea^15^. Using the manual of operation (MOP), the KNN data are systematically collected and recorded at various time points, including during NICU hospitalization, at discharge, and a CA of 18–24 months via electronic case report forms within the internet-based clinical trial management system administered by the KNIH. The KNN registry data undergo annual storage in the KNIH server after rigorous data quality management procedures, including queries and site-visit monitoring^15^. The Institutional Review Board at each participating NICU approved the KNN registry, and informed consent was obtained from the parents upon enrollment of their infants in KNN-affiliated NICUs. All research methods complied with the relevant guidelines and regulations.

### Study population

**Figure 1:**
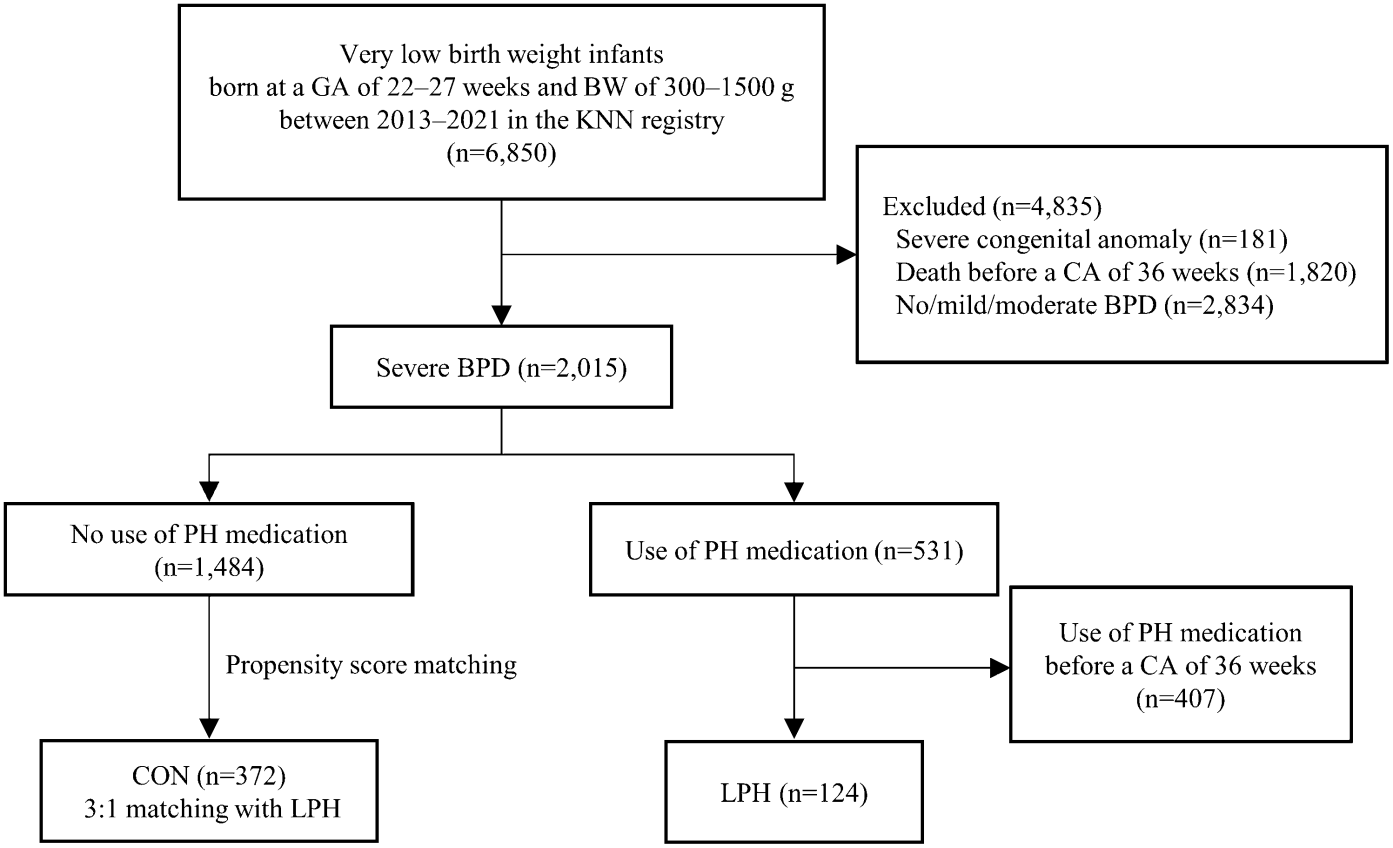
Flow chart of the study population. *GA* gestational age, *BW* birthweight, *KNN* Korean Neonatal Network, *CA* corrected age, *BPD* bronchopulmonary dysplasia, *PH* pulmonary hypertension.

Initially, we collected 6,850 VLBWIs with 22–27 weeks of gestation and birthweight of 300–1500 g born between January 2013 and December 2021. Among them, infants with severe congenital anomaly (n = 181), who died before a CA of 36 weeks (n = 1820), or who were classified into no/mild/moderate BPD (n = 2834) were excluded. Of the remaining 2015 EPIs who survived at a CA of 36 weeks having severe BPD, 407 infants who were diagnosed as PH and received initial PH medication before a CA of 36 weeks were also excluded. Finally, we enrolled 1608 EPIs with severe BPD comprising two groups: 1484 infants without PH medication (no-PH) and 124 infants who were diagnosed as late PH requiring PH medication after a CA of 36 weeks (LPH) (Fig. 1). LPH was matched 1:3 with no-PH as controls (CON, n = 372) using propensity score matching (PSM) within demographic characteristics including gestational age (GA), cesarean section (C/S), birthweight, and year of birth, which significantly differed between the two groups (Supplementary Table 1).

### Definitions

BPD diagnosis and grading were based on the National Institute of Child Health and Human Development 2001 definition with a precondition of oxygen or respiratory support in at least the first 28 days of life and assessed at a CA of 36 weeks^16^, according to the KNN’s MOP.

We defined infants with late PH as those who were diagnosed as PH with the initiation of PH medication after a CA of 36 weeks^17^. Detailed echocardiographic findings were unavailable; however, the KNN’s MOP defines PH as confirmed or suspected based on echocardiography or clinical assessment, prompting initiation of PH medication. Information on PH medication is selected from options including inhaled nitric oxide (NO), sildenafil, iloprost, bosentan, milrinone, and other medications through manual entry. At the same time, the date of the first administration of PH medication is inputted through a calendar-click structure. In most KNN-affiliated NICUs, if infants are diagnosed with severe BPD at a CA of 36 weeks, regular echocardiographic evaluation is routinely performed within 2 weeks followed by monthly surveillance thereafter for the surveillance of late PH. Furthermore, to qualify for Korean national medical insurance coverage of medications used for PH, echocardiographic results are typically required for submission. Therefore, the diagnosis of PH was mainly based on echocardiography. Therefore, in the present study, we enrolled the LPH cases diagnosed as PH with initiating PH medication after a CA of 36 weeks.

### Data collection

Demographics including maternal/neonatal characteristics, short-term outcomes, and long-term outcomes at a CA of 18–24 months, were collected.

Maternal characteristics included maternal age, multiple gestations, gestational diabetes, maternal hypertension, C/S, preterm premature rupture of membranes ≥ 24 h, oligohydramnios, use of antenatal steroids, and pathologic chorioamnionitis. Neonatal characteristics included GA, birthweight and height, small for gestational age (SGA) (< 10th percentile by Fenton growth chart)^18^, sex, Apgar scores at 1 and 5 min, duration of supplemental O_2_ and non-invasive/invasive ventilator, and use of steroids for BPD. Respiratory distress syndrome, pneumothorax, culture-proven sepsis, patent ductus arteriosus (PDA) needing pharmacological or surgical treatment, intraventricular hemorrhage (IVH; ≥ grade III according to the Papile classification), periventricular leukomalacia (PVL), necrotizing enterocolitis (≥ stage II according to the Bell’s criteria), retinopathy of prematurity (≥ stage 3 or needing operation), need for home oxygen at discharge, length of hospitalization, and mortality during NICU hospitalization were also collected as neonatal short-term outcomes.

Long-term outcomes at a CA of 18–24 months included present data for home oxygen use; PH medications; rehabilitation; speech treatment; re-hospitalization; NDD assessed using Bayley Scales of Infant Development (BSID) II & III, Korean Developmental Screening Test for infants and children (K-DST), or Korean version of the Ages and Stages Questionnaire (K-ASQ); cerebral palsy (Gross Motor Function Classification System ≥ 2)^19^; blindness or wearing glasses; hearing loss (use of hearing aid or cochlear implant); growth outcomes (Z-scores of body weight, height, and head circumference according to the 2006 World Health Organization (WHO) Child Growth Standards^20^) and postnatal growth failure (PGF); and long-term mortality after a CA of 36 weeks.

### Outcomes

The primary outcome was long-term mortality after a CA of 36 weeks or NDD confirmed at a CA of 18–24 months. The secondary outcomes were long-term mortality and other neurodevelopmental outcomes, such as each domain of NDD, cerebral palsy, blindness or wearing glasses, hearing loss, and growth outcomes including PGF at a CA of 18–24 months.

Long-term mortality was defined as mortality from a CA of 36 weeks until a CA of 18–24 months.

We defined NDD by classifying it into distinct domains based on results from all available tests, specifically mental, motor, and social domains as follows^21–25^: mental domain NDD by the combination of results from mental developmental index of BSID-II, cognition or language of BSID-III, cognition, language, or self-help of K-DST, or communication or problem-solving of K-ASQ; motor domain NDD from the psychomotor developmental index of BSID-II, motor scale of BSID-III, gross or fine motor of K-DST or K-ASQ; social domain NDD from social interaction of K-DST or K-ASQ. Although K-DST or K-ASQ is highly correlated with BSID^22–25^, we regarded BSID-II or III as a confirmatory test and K-ASQ or K-DST as a screening test for NDD. Therefore, NDD in each domain was defined primarily when the BSID scores were < 70 regardless of screening results from K-DST or K-ASQ and, secondarily when at least one of the scores in a specific domain from K-DST or K-ASQ was < − 2 standard deviations (SD) if BSID scores were missing^26^. If any of the three domains was abnormal, it was classified as NDD for the overall domain^26–28^.

PGF was defined as body weight, height, or head circumference that did not exceed Z-score of − 2 (2.3rd percentile) according to the WHO Child Growth Standards^20,26^.

### Statistical analysis

PSM was conducted for a comparison between no-PH and LPH. Comparisons were made between CON and LPH, between infants without and with a primary outcome, and between infants with and without follow-up loss for long-term neurodevelopmental tests.

Categorical variables were presented as numbers and percentages and analyzed using the chi-squared or Fisher’s exact test. After performing a Shapiro–Wilk normality test, continuous variables were presented as means ± SD and analyzed using the* t*-test or Mann–Whitney U test.

Multivariable logistic regression analysis was performed to investigate any independent association between late PH and adverse effects on primary outcome with covariates, which presented significant differences between infants without and with primary outcome. The adjusted odds ratio (aOR) with 95% confidence intervals (CI) is presented, and statistical significance was set at *P*-value < 0.05. All statistical analyses were performed using STATA version 16.0 (StataCorp, College Station, TX, USA).

## Results

### Clinical characteristics

Of pre-enrolled 2015 EPIs with severe BPD, 531 (26.4%) and 124 (6.2%) were diagnosed as PH and received PH medications during NICU hospitalization at any time and after a CA of 36 weeks, respectively (Fig. 1). Different medications were used for late PH treatment: 83 cases (66.9%) used only one medication, while 41 cases (33.1%) used two or more medications (Table 1). The most frequently used medications were sildenafil (n = 97), inhaled NO (n = 40), and bosentan (n = 19), in that order.

**Table 1:** PH medications used for late PH treatment in the study.

Late PH medications	Numbers
Sildenafil	64
Inhaled NO	13
Bosentan	3
Iloprost	1
Beraprost	2
Sildenafil + Inhaled NO	11
Sildenafil + Bosentan	5
Sildenafil + Iloprost	3
Sildenafil + Milrinone	1
Inhaled NO + Iloprost	1
Inhaled NO + Treprostinil	1
Inhaled NO + Milrinone	4
Bosentan + Iloprost	1
Sildenafil + Inhaled NO + Bosentan	2
Sildenafil + Inhaled NO + Milrinone	3
Sildenafil + Bosentan + Iloprost	2
Sildenafil + Bosentan + Milrinone	1
Inhaled NO + Iloprost + Milrinone	1
Sildenafil + Inhaled NO + Bosentan + Iloprost	1
Sildenafil + Inhaled NO + Bosentan + Beraprost	1
Sildenafil + Inhaled NO + Bosentan + Milrinone	1
Sildenafil + Bosentan + Iloprost + Milrinone	1
Sildenafil + Inhaled NO + Bosentan + Iloprost + Treprostinil	1
	Total = 124

*PH* pulmonary hypertension, *NO* nitric oxide.

The comparisons of demographic characteristics and short-term outcomes between CON and LPH and between infants without and with a primary outcome are detailed in Table 2. Both univariate analyses showed no significant differences in maternal characteristics. However, neonatal short-term outcomes, including duration of invasive (*P* < 0.001) and non-invasive ventilator (*P* = 0.015), use of steroids for BPD (*P* = 0.025), PDA treated with operation (*P* = 0.005), need for home oxygen at discharge (*P* = 0.004), length of hospitalization (*P* < 0.001), and mortality in NICU after a CA of 36 weeks (*P* < 0.001) were significantly higher in LPH than in CON. In contrast, PDA treated with medication (*P* = 0.041) was lower in LPH than in CON.

**Table 2:** Comparisons of demographic characteristics and short-term outcomes (CON vs. LPH and No primary outcome vs. Primary outcome).

	CON (n = 372)	LPH (n = 124)	*P*-value	No long-term mortality or NDD (n = 354)	Long-term mortality or NDD (n = 142)	*P*-value
**Maternal characteristics**						
Maternal age, years	33.6 (3.9)	34.1 (4.1)	0.321	33.8 (3.9)	33.6 (4.0)	0.571
Gestational diabetes	23/372 (6.2)	11/124 (8.9)	0.305	28/354 (7.9)	6/142 (4.2)	0.142
Maternal hypertension	72/372 (19.4)	22/124 (17.7)	0.691	65/354 (18.4)	29/142 (20.4)	0.597
Multiple gestations	124/372 (33.3)	42/124 (33.9)	0.913	120/354 (33.9)	46/142 (32.4)	0.748
Cesarean section	242/372 (65.1)	83/124 (66.9)	0.703	238 /354 (67.2)	87/142 (61.3)	0.207
PPROM ≥ 24 h	95/122 (77.9)	29/42 (69.1)	0.251	96/127 (75.6)	28/37 (75.7)	0.992
Oligohydramnios	58/332 (17.5)	20/116 (17.2)	0.955	58/321 (18.1)	20/127 (15.8)	0.559
Use of antenatal steroids	319/364 (87.6)	99/118 (83.9)	0.298	305/348 (87.6)	113/134 (84.3)	0.337
Pathologic chorioamnionitis	167/335 (49.9)	50/105 (47.6)	0.690	152/316 (48.1)	65/124 (52.4)	0.415
**Neonatal characteristics**						
Gestational age, week	25.1 (1.4)	25.0 (1.4)	0.672	25.1 (1.4)	25.0 (1.4)	0.751
Birth weight, g	725.8 (182.8)	720.9(187.8)	0.767	740.2 (180.3)	685.6 (187.6)	0.003*
Birth height, cm	32.0 (3.0)	31.6 (2.8)	0.193	32.2 (2.8)	31.2 (3.1)	0.001*
Small for gestational age	67/368 (18.2)	25/123 (20.3)	0.602	53/351 (15.1)	39/140 (27.9)	0.001*
Males	186/372 (50.0)	63/123 (51.2)	0.815	182/353 (51.6)	67/142 (47.2)	0.379
1 min Apgar score	3.6 (1.7)	3.9 (1.8)	0.097	3.7 (1.7)	3.7 (1.7)	0.866
5 min Apgar score	6.0 (1.7)	6.1 (1.8)	0.581	6.1 (1.7)	5.9 (1.9)	0.301
Supplemental O_2_ days	12.3 (14.9)	18.7 (26.8)	0.112	14.0 (16.4)	13.7 (23.7)	0.065
Invasive ventilator days	52.5 (30.1)	101.7 (78.0)	< 0.001*	55.5 (41.5)	87.9 (65.1)	< 0.001*
Non-invasive ventilator days	45.1 (25.5)	59.9 (49.7)	0.015*	48.3 (27.6)	50.0 (45.9)	0.229
Use of steroids for BPD	286/372 (76.9)	107/124 (86.3)	0.025*	283 /354 (79.9)	110/142 (77.5)	0.538
Respiratory distress syndrome	366/372 (98.4)	119/124 (96.0)	0.113	349/354 (98.6)	136/142 (95.8)	0.054
Pneumothorax	31/372 (8.3)	13/124 (10.5)	0.466	28/354 (7.9)	16/142 (11.3)	0.234
Late pulmonary hypertension				68/354 (19.2)	56/142 (39.4)	< 0.001*
Culture proven sepsis	190/372 (51.1)	62/124 (50.0)	0.836	171 /354 (48.3)	81/142 (57.0)	0.079
PDA, medication	218 /282 (77.3)	62/93 (66.7)	0.041*	198/262 (75.6)	82/113 (72.6)	0.539
PDA, operation	125/ 282 (44.3)	57/93 (61.3)	0.005*	120/262 (45.8)	62/113 (54.9)	0.107
IVH ≥ grade III	83/372 (22.3)	29/124 (23.4)	0.804	62/354 (17.5)	50/142 (35.2)	< 0.001*
Periventricular leukomalacia	62/371 (16.7)	30/123 (24.4)	0.058	51/353 (14.5)	41/141 (29.1)	< 0.001*
NEC ≥ stage II	55/372 (14.8)	25/124 (20.2)	0.159	50/354 (14.1)	30/142 (21.1)	0.055
ROP, operation	136/316 (43.0)	44/102 (43.1)	0.986	120/297 (40.4)	60/121 (49.6)	0.086
ROP ≥ Stage III	172/369 (46.6)	59/124 (47.6)	0.852	159/353 (45.0)	72/140 (51.4)	0.200
Need for home oxygen at discharge	34/315 (10.8)	14/57 (24.6)	0.004*	37/289 (12.8)	11/83 (13.3)	0.914
Length of hospital days	129.7 (37.5)	192.7 (79.0)	< 0.001*	136.9 (46.8)	166.6 (75.1)	0.001*
Mortality during NICU (after a CA of 36 weeks)	13/372 (3.5)	23/124 (18.6)	< 0.001*			

Categorical variables are presented as N (%). Continuous variables are presented as means (standard deviation). *NDD* neurodevelopmental delay, *PPROM* preterm premature rupture of membranes, *BPD* bronchopulmonary dysplasia, *PDA* patent ductus arteriosus, *IVH* intraventricular hemorrhage, *NEC* necrotizing enterocolitis, *ROP* retinopathy of prematurity, *NICU* neonatal intensive care unit, *CA* corrected age. * *P*-value < 0.05.

Infants with a primary outcome had lower birthweight (*P* = 0.003) and height (*P* = 0.001), higher incidence of SGA (*P* = 0.001), late PH (*P* < 0.001), IVH ≥ grade III (*P* < 0.001), and PVL (*P* < 0.001) as well as longer duration of the invasive ventilator (*P* < 0.001) and length of hospitalization (*P* < 0.001) than those without primary outcome.

### Long-term mortality and other outcomes

Long-term mortality after a CA of 36 weeks and other outcomes at a CA of 18–24 months between CON and LPH are presented in Table 3. Among enrolled 496 infants, a total of 460 infants survived (359 in CON and 101 in LPH) at NICU discharge with the exclusion of 36 deaths (13 in CON and 23 in LPH) in NICU, and 288 (62.6%) of 460 infants with 232 (64.6%) in CON and 56 (55.4%) in LPH were followed up at a CA of 18–24 months. Among the infants having long-term follow-up data, 77.4% were assessed using neurodevelopmental tests (79.7% in CON and 67.9% in LPH). We investigated whether there were differences in demographics and short-term outcomes among enrolled infants based on the follow-up status of neurodevelopmental tests (Supplementary Table 2). In CON, infants who underwent neurodevelopmental testing presented a higher incidence of maternal pathologic chorioamnionitis and severe ROP compared to those who lost follow-up. In contrast, in LPH, there were no significant differences between infants with follow-up loss and those without follow-up loss for neurodevelopmental testing.

**Table 3:** Comparison of mortality after a CA of 36 weeks and long-term outcomes at a CA of 18–24 months (CON vs. LPH).

	CON (n = 372)^#^	LPH (n = 124)^#^	*P*-value
**Primary outcome**			
Long-term mortality or NDD	86/372 (23.1)	56/124 (45.2)	< 0.001*
**Secondary outcomes**			
Long-term mortality (after a CA of 36 weeks)^a^	18/372 (4.8)	30/124 (24.2)	< 0.001*
Mortality during NICU	13	23	
Mortality after discharge	5	7	
NDD for the overall domain	70/185 (37.8)	26/38 (68.4)	0.001*
Mental domain	56/185 (30.3)	22/38 (57.9)	0.001*
Motor domain	50/185 (27.0)	19/38 (50.0)	0.005*
Social domain	25/119 (21.0)	6/26 (23.1)	0.816
Cerebral palsy	28/ 217 (12.9)	10/53 (18.9)	0.263
Blindness	8 /222 (3.6)	1/49 (2.0)	0.989
Wearing glasses	16/224 (7.1)	3/51 (5.9)	0.999
Hearing loss	10/226 (4.4)	1/53 (1.9)	0.696
Weight (Z-score)	− 0.45 (1.0)	− 0.54 (0.9)	0.539
Height (Z-score)	− 0.37 (1.0)	− 0.63 (0.9)	0.110
Head circumference (Z-score)	− 0.53 (1.1)	− 0.77 (1.0)	0.145
Postnatal growth failure (Z-score < − 2.0)			
Weight	9/224 (4.0)	3/53 (5.7)	0.597
Height	11/213 (5.2)	4/50 (8.0)	0.437
Head circumference	17/178 (9.6)	4/43 (9.3)	0.960
**Others**			
Use of home oxygen	1/155 (0.7)	1/27 (3.7)	0.275
Use of PH medications	1/109 (0.9)	4/22 (18.2)	< 0.001*
Rehabilitation treatment	100/176 (56.8)	34 /45 (75.6)	0.022*
Speech treatment	34/216 (15.7)	15/51 (29.4)	0.023*
Re-hospitalization after discharge	144/226 (63.7)	41/54 (75.9)	0.089

Categorical variables are presented as N (%). Continuous variables are presented as means (standard deviation). *CA* corrected age, *PH* pulmonary hypertension, *NDD* neurodevelopmental delay.

^a^Mortality from a CA of 36 weeks until a CA of 18–24 months.

*P-value < 0.05.

^#^The number of long-term follow-ups at a CA of 18–24 months are 232 and 56 in CON and LPH, respectively.

Nevertheless, long-term mortality or NDD as the primary outcome was significantly higher in LPH than in CON (*P* < 0.001). As secondary outcomes, long-term mortality was significantly higher (*P* < 0.001), along with higher NDD prevalence (*P* = 0.001) in LPH than in CON, mainly in the mental (*P* = 0.001) and motor domain (*P* = 0.005). The Z-scores of body gauge including body weight, height, and head circumference at a CA of 18–24 months did not significantly differ between CON and LPH, and there were also no differences in PGF rate. In addition, there were no differences in other neurodevelopmental outcomes such as cerebral palsy, blindness or wearing glasses, and hearing loss between CON and LPH. However, the present PH medication use (*P* < 0.001), rehabilitation (*P* = 0.022), and speech treatment (*P* = 0.023) were significantly more frequent in LPH than in CON.

### Risk factors for primary outcome

**Figure 2:**
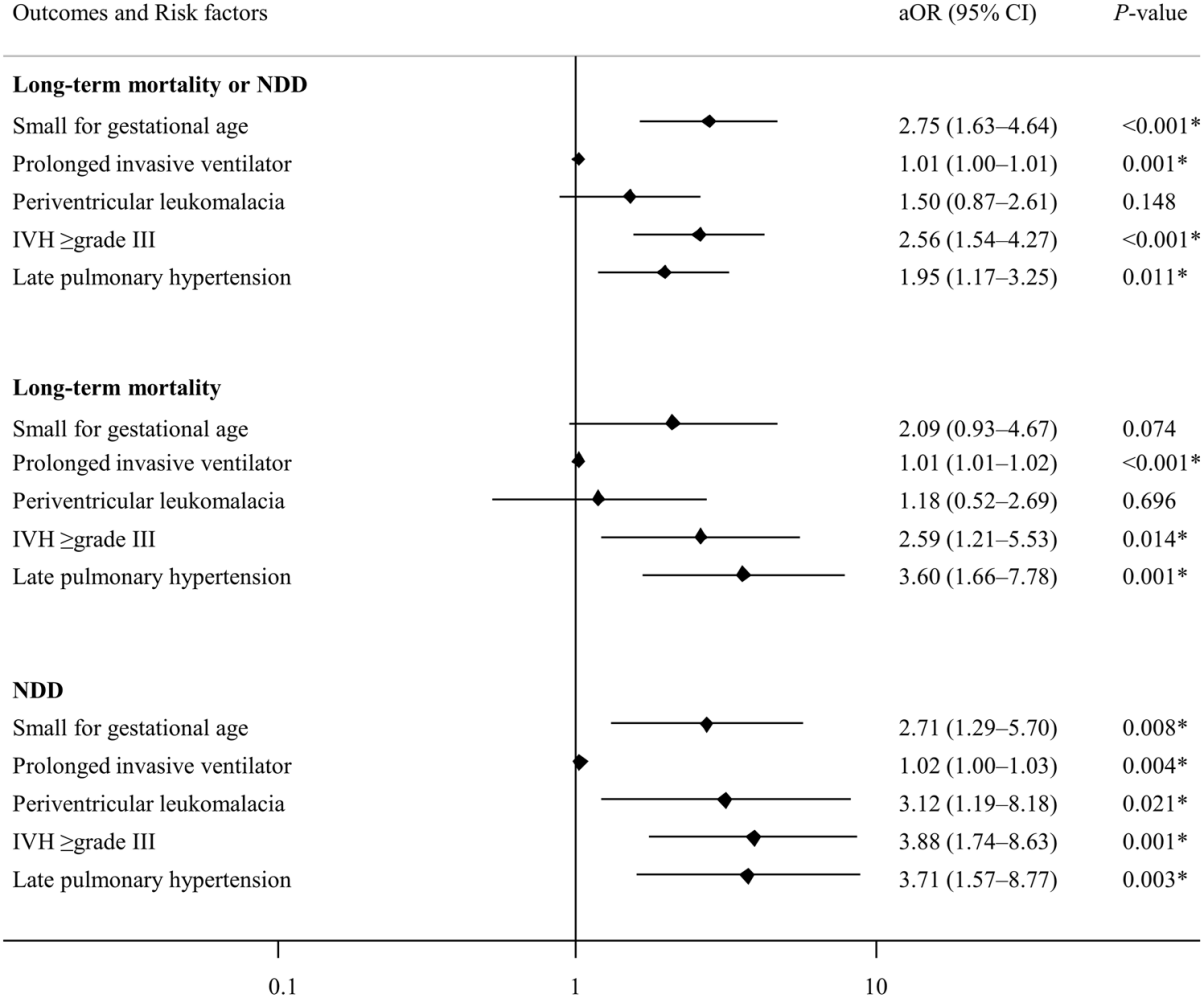
Multivariable regression analysis for risk factors associated with primary outcome. *NDD* neurodevelopmental delay, *aOR* adjusted odds ratio, *CI* confidence intervals, *IVH* intraventricular hemorrhage.

Multivariable regression analyses were done with adjusting for variables found as the significant factors in univariate analysis for the primary outcome, including SGA, prolonged invasive ventilator, PVL, IVH ≥ grade III, and late PH (Fig. 2). The results revealed SGA (aOR, 2.75; 95% CI, 1.63–4.64), prolonged invasive ventilator (aOR, 1.01, 95% CI, 1.00–1.01), and IVH ≥ grade III (aOR, 2.56; 95% CI, 1.54–4.27), as well as late PH (aOR, 1.95; 95% CI, 1.17–3.25) independently increased the risk of the primary outcome of long-term mortality or NDD. Late PH also increased the risk of long-term mortality (aOR, 3.60; 95% CI, 1.66–7.78) and of NDD (aOR, 3.71; 95% CI, 1.57–8.77) respectively.

## Discussion

The present study has demonstrated that late PH requiring PH medication independently increased the risk of long-term mortality or NDD in EPIs with severe BPD. To correct the impact of BPD itself or its severity on neurodevelopment^3–6^, only EPIs with severe BPD were included as the study population. Therefore, we could clarify the apparent adverse impact of late PH on long-term outcomes in EPIs.

As the LPH group exhibited greater disease severity, such as prolonged ventilation, hospitalization, and home oxygen use, those results raise concern about residual confounding, which might suggest that the observed association with worse long-term outcomes in LPH may be partially due to underlying illness severity rather than a true association. However, we tried to mitigate that concern through multivariable regression analysis after additional analysis between infants without and with a primary outcome.

Despite increased survival and follow-up rates in EPIs, information regarding the long-term outcomes of those with late PH is relatively lacking. There was only a sparse accumulation of data on infants surviving with late PH, which showed the adverse effects of late PH on long-term outcomes^13,14^. Nakanishi et al., reported in their single-center study that developmental delay according to Kyoto Scale of Psychological Development (KSPD) and growth restriction of body weight were more severe in EPIs with BPD-associated PH than in those with BPD alone at a CA of 3 years. Their multiple regression analysis demonstrated that BPD-associated PH was an independent risk factor for developmental delay along with PVL^13^. Another single-center study has also reported that EPIs with BPD-associated PH had poorer developmental scores according to BSID-III and poorer growth than those with BPD only at a CA of 18–24 months^14^. In those two retrospective studies, the neurodevelopmental assessment was implemented using one modality (either KSPD or BSID-III), and they only included a small number of infants having late PH (22 and 20, respectively) without delineation of BPD severity owing to the single-center design. One multi-center study reported that infants with severe BPD born at a GA < 32 weeks had a high re-admission rate at a CA of 1 year when affected by PH without presenting any information on neurodevelopmental outcomes^12^.

Therefore, one of the strengths of this study is that it utilizes a multi-center prospective cohort to assess the association between late PH requiring PH medication and long-term outcomes, including survival and neurodevelopment of EPIs with severe BPD.

In our study, the Z-scores and PGF rate of body weight, height, and head circumference at a CA of 18–24 months did not show significant differences between CON and LPH. This contrasts with previous studies indicating an association between late PH and growth restriction^13,14^. The lack of significant differences in our study may be attributed to PSM, which adjusted for GA and birthweight, minimizing variations in perinatal factors between the two groups. This implies that when the SGA component is accounted for, late PH might not independently contribute to long-term growth failure in EPIs.

As indicated in various studies, late PH associated with severe BPD manifests as severe pulmonary vascular disease^29,30^, leading to right ventricular dysfunction and an increased risk of progressing to right heart failure, which is a significant predictor of mortality^31^. Additionally, enduring morbidities linked to this condition, including persistent hypoxemia, prolonged ventilator dependence, and the need for sustained caloric support, are likely to adversely impact brain development and growth, ultimately resulting in unfavorable neurodevelopmental outcomes.

The present study has several limitations. First, concerning the diagnosis of late PH, we defined late PH according to the KNN's MOP, which indicates that PH is confirmed or suspected based on echocardiography or clinical assessment, prompting initiation of PH medication after a CA of 36 weeks. Consequently, we could not analyze precise echocardiographic findings. Since echocardiography is the gold standard for diagnosing PH and a screening echocardiogram would be conducted in most units, especially in severe BPD cases^9,32–35^, the initiation of PH medication was based on the attending clinician’s decision, guided by echocardiographic results. The PH medications mainly used in our study (sildenafil, inhaled NO, and bosentan) are generally used in clinical practice^7,9^. Therefore, these diagnostic criteria in the present study are valuable as real-world data. However, there is a limitation in that cases without medication for PH diagnosed by echocardiogram could be excluded. Additionally, in cases medicated at least once in early life, they are also excluded even if they resumed medication after a CA of 36 weeks. This exclusion may result in an underestimation of the diagnosis of late PH, especially considering that early PH is a significant risk factor for BPD-associated late PH^33^. Conversely, it should also be noted as a limitation that there are a few possibilities of including cases where inhaled NO was used for improving ventilation/perfusion (V/Q) mismatch even in infants with severe BPD who were not diagnosed with PH. Also, it should be considered a limitation that the absence of echocardiographic findings precluded the possibility of the impact of the degree of PH on long-term outcomes. Additionally, due to the nature of the KNN registry, there is insufficient data on each patient's status upon administration of PH medication, making it challenging to analyze and provide suggestions regarding the effects of medication use.

Second, because of the multi-center registry nature, various neurodevelopmental assessment modalities were used in this study. To use all available data to mitigate selection bias, we developed a composite score by combining BSID-II & III as confirmatory tests and K-DST and K-ASQ as screening tests^26^. Despite these efforts, it is crucial to acknowledge the potential presence of bias due to inherent inter-test variability. While questionnaires like K-DST/K-ASQ have been reported to be valid and correlate with BSID^22–25^, there may still be recall bias. Although we used the same cut-off value (< 70 for NDD) for both BSID-II and III as in previous studies^26–28^, controversies persist regarding the use of various cut-off values for them^21,36,37^.

Lastly, a potential limitation of the current study is that a substantial number of infants were lost to long-term follow-up. The registry-based analysis resulted in particularly significant follow-up loss for neurodevelopmental assessment in our study population. While results of neurodevelopmental tests are not mandatory and are entered based on individual hospital practices, missing data can occur even with follow-up and collection of other items if the test is not performed. However, when comparing demographics and short-term outcomes of each group according to the follow-up status of neurodevelopmental tests, no significant factors were found to induce follow-up loss in either group. Consequently, there were no specific circumstances which lead to a potential underestimation or overestimation of the adverse impact of late PH on long-term outcomes in the present study.

Nevertheless, this study's strength lies in its inclusion of a large number of EPIs with late PH using a national prospective cohort from multi-centers and investigation of their actual long-term clinical course, including neurodevelopmental aspects, at a national level.

## Conclusion

Late PH requiring medication independently increased the risk of long-term mortality or NDD at a CA of 18–24 months in EPIs with severe BPD, along with a similar impact on mortality and NDD, respectively. Therefore, it is crucial to maintain ongoing vigilant monitoring and meticulous care for the improved long-term survival and neurodevelopment of EPIs with severe BPD and late PH, even after NICU discharge. Future studies that employ standardized criteria including echocardiographic assessments for PH diagnosis and include infants without BPD, are essential to validate the lasting effects of late PH on EPIs.

### Supplementary Information

Supplementary Tables.

## Data Availability

The data that support the findings of this study are available from the corresponding author (yschang@skku.edu) upon reasonable request.
