# Supplementary material for: Long-term impact of late pulmonary hypertension requiring medication in extremely preterm infants with severe bronchopulmonary dysplasia
Source: Sci Rep. 2024 Apr 15;14:8705. doi: 10.1038/s41598-024-58977-w (PMC11018761; doi:10.1038/s41598-024-58977-w)
Supplement: Supplementary file 1 — Supplementary Tables. [file 41598_2024_58977_MOESM1_ESM.docx]

**Supplementary Table 1.** Comparison of demographic characteristics and short-term outcomes (No-PH vs. LPH). Categorical variables are presented as N (%). Continuous variables are presented as means (standard deviation). *PPROM* preterm premature rupture of membranes, *BPD* bronchopulmonary dysplasia, *PDA* patent ductus arteriosus, *IVH* intraventricular hemorrhage, *NEC* necrotizing enterocolitis, *ROP* retinopathy of prematurity, *NICU* neonatal intensive care unit, *CA* corrected age. * *P*-value <0.05.

|  | **No-PH (n=1,484)** | **LPH (n=124)** | ***P*-value** | |
| --- | --- | --- | --- | --- |
| **Maternal characteristics** | | | |  |
| Maternal age, y | 33.4 (4.2) | 34.1 (4.1) | 0.074 | |
| Gestational diabetes | 113/1,484 (7.6) | 11/124 (8.9) | 0.614 | |
| Maternal hypertension | 221/1,484 (14.9) | 22/124 (17.7) | 0.395 | |
| Multiple gestations | 502/1,484 (33.8) | 42/124 (33.9) | 0.992 | |
| Cesarean section | 1,133/1,484 (76.4) | 83/124 (66.9) | 0.019* | |
| PPROM ≥24 h | 378/ 496 (76.2) | 29/42 (69.1) | 0.299 | |
| Oligohydramnios | 211/1,346 (15.7) | 20/116 (17.2) | 0.657 | |
| Use of antenatal steroids | 1,272/1,460 (87.1) | 99/118 (83.9) | 0.318 | |
| Pathologic chorioamnionitis | 644/1,305 (49.4) | 50/105 (47.6) | 0.733 | |
| **Neonatal characteristics** | | | |  |
| Gestational age, wk | 25.4 (1.3) | 25.0 (1.4) | 0.003* | |
| Birth weight, g | 812.6 (187.9) | 720.9 (187.8) | <0.001* | |
| Birth height, cm | 33.2 (2.8) | 31.6 (2.8) | <0.001* | |
| Small for gestational age | 146/1,484 (9.8) | 25/123 (20.3) | <0.001* | |
| Males | 681/1,484 (45.9) | 63/123 (51.2) | 0.255 | |
| 1 min Apgar score | 3.7 (1.7) | 3.9 (1.8) | 0.296 | |
| 5 min Apgar score | 6.1 (1.7) | 6.1 (1.8) | 0.871 | |
| Supplemental O_2_ days | 12.2 (17.3) | 18.7 (26.8) | 0.017* | |
| Invasive ventilator days | 47.4 (31.9) | 101.7 (78.0) | <0.001* | |
| Non-invasive ventilator days | 44.7 (24.8) | 59.9 (49.7) | 0.005* | |
| Use of steroids for BPD | 1,081/1,484 (72.8) | 107/124 (86.3) | 0.001* | |
| Respiratory distress syndrome | 1,457/1,484 (98.2) | 119/124 (96.0) | 0.090 | |
| Pneumothorax | 103/1,484 (6.9) | 13/124 (10.5) | 0.143 | |
| Culture proven sepsis | 620/1,484 (41.8) | 62/124 (50.0) | 0.075 | |
| PDA, medication | 827/1,107 (74.7) | 62/93 (66.7) | 0.089 | |
| PDA, operation | 465/1,107 (42.0) | 57/93 (61.3) | <0.001* | |
| IVH ≥grade III | 262/1,484 (17.7) | 29/124 (23.4) | 0.111 | |
| Periventricular leukomalacia | 227/1,483 (15.3) | 30/123 (24.4) | 0.008* | |
| NEC ≥stage Ⅱ | 210/1,484 (14.2) | 25/124 (20.2) | 0.069 | |
| ROP, operation | 442/1,199 (36.9) | 44/102 (43.1) | 0.209 | |
| ROP ≥stage III | 605/1,475 (41.0) | 59/124 (47.6) | 0.154 | |
| Need for home oxygen at discharge | 130/1,249 (10.4) | 14/57 (24.6) | 0.001* | |
| Length of hospital days | 124.0 (36.7) | 192.7 (79.0) | <0.001* | |
| Mortality during NICU  (after a CA of 36 weeks) | 45/1,484 (3.0) | 23/124 (18.6) | <0.001* | |

**Supplementary Table 2.** Comparison of demographic characteristics and short-term outcomes (ND F/U loss vs. ND F/U). Categorical variables are presented as N (%). Continuous variables are presented as means (standard deviation). *ND* neurodevelopmental. *F/U* follow-up, *PPROM* preterm premature rupture of membranes, *BPD* bronchopulmonary dysplasia, *PDA* patent ductus arteriosus, *IVH* intraventricular hemorrhage, *NEC* necrotizing enterocolitis, *ROP* retinopathy of prematurity. * *P*-value <0.05.

|  | **CON (n=359)** | | | **LPH (n=101)** | | |
| --- | --- | --- | --- | --- | --- | --- |
|  | **ND F/U loss (n=174)** | **ND F/U (n=185)** | ***P*-value** | **ND F/U loss (n=63)** | **ND F/U (n=38)** | ***P*-value** |
| **Maternal characteristics** | | | | | | |
| Maternal age, y | 33.3 (4.0) | 33.9 (3.8) | 0.237 | 33.9 (4.4) | 34.2 (4.3) | 0.877 |
| Gestational diabetes | 11/174 (6.3) | 12/185 (6.5) | 0.949 | 8/63 (12.7) | 3/38 (7.9) | 0.528 |
| Maternal hypertension | 33/174 (19.0) | 35/185 (18.9) | 0.991 | 11/63 (17.5) | 8/38 (21.1) | 0.654 |
| Multiple gestations | 54/174 (31.0) | 64/185 (34.6) | 0.473 | 27/63 (42.9) | 10/38 (26.3) | 0.095 |
| Cesarean section | 117/174 (67.2) | 114/185 (61.6) | 0.267 | 42/63 (66.7) | 29/38 (76.3) | 0.304 |
| PPROM ≥24 h | 48/67 (71.6) | 43/51 (84.3) | 0.105 | 14/22 (63.6) | 13/17 (76.5) | 0.389 |
| Oligohydramnios | 23/152 (15.1) | 33/170 (19.4) | 0.312 | 13/58 (22.4) | 7/37 (18.9) | 0.684 |
| Use of antenatal steroids | 152/172 (88.4) | 155/179 (86.6) | 0.615 | 51/62 (82.3) | 32/36 (88.9) | 0.562 |
| Pathologic chorioamnionitis | 69/156 (44.2) | 93/168 (55.4) | 0.045* | 23/50 (46.0) | 19/37 (51.4) | 0.621 |
| **Neonatal characteristics** | | | | | | |
| Gestational age, wk | 25.2 (1.4) | 25.0 (1.5) | 0.532 | 24.9 (1.5) | 25.3 (1.2) | 0.261 |
| Birth weight, g | 748.3 (185.3) | 710.8 (177.3) | 0.121 | 727.5 (170.4) | 723.3 (210.7) | 0.847 |
| Birth height, cm | 32.5 (2.7) | 31.7 (3.1) | 0.040* | 31.8 (2.3) | 31.2 (3.5) | 0.345 |
| Small for gestational age | 24/173 (13.9) | 38/182 (20.9) | 0.082 | 7/62 (11.3) | 10/38 (26.3) | 0.052 |
| Males | 94/174 (54.0) | 86/185 (46.5) | 0.153 | 27/62 (43.6) | 21/38 (55.3) | 0.255 |
| 1 min Apgar score | 3.6 (1.7) | 3.6 (1.6) | 0.653 | 3.8 (1.7) | 4.2 (1.7) | 0.374 |
| 5 min Apgar score | 6.0 (1.7) | 6.1 (1.7) | 0.792 | 6.0 (1.6) | 6.4 (1.9) | 0.160 |
| Supplemental O_2_ days | 12.3 (14.8) | 12.9 (15.1) | 0.490 | 20.7 (25.3) | 23.0 (33.1) | 0.954 |
| Invasive ventilator days | 50.8 (30.4) | 51.7 (28.7) | 0.807 | 105.8 (84.9) | 78.1 (62.4) | 0.100 |
| Non-invasive ventilator days | 46.0 (26.5) | 46.0 (22.3) | 0.888 | 63.8 (54.3) | 66.1 (47.5) | 0.666 |
| Use of steroids for BPD | 132/174 (75.9) | 144/185 (77.8) | 0.657 | 57/63 (90.5) | 31/38 (81.6) | 0.196 |
| Respiratory distress syndrome | 171/174 (98.3) | 182/185 (98.4) | 0.940 | 62/63 (98.4) | 34/38 (89.5) | 0.065 |
| Pneumothorax | 11/174 (6.3) | 17/185 (9.2) | 0.311 | 5/63 (7.9) | 5/38 (13.2) | 0.395 |
| Culture proven sepsis | 88/174 (50.6) | 92/185 (49.7) | 0.873 | 33/63 (52.4) | 14/38 (36.8) | 0.129 |
| PDA, medication | 100/130 (76.9) | 111/142 (78.2) | 0.806 | 32/49 (65.3) | 18/24 (75.0) | 0.402 |
| PDA, operation | 56/130 (43.1) | 66/142 (46.5) | 0.573 | 31/49 (63.3) | 15/24 (62.5) | 0.949 |
| IVH ≥grade III | 36/174 (20.7) | 41/185 (22.2) | 0.734 | 15/63 (23.8) | 6/38 (15.8) | 0.336 |
| Periventricular leukomalacia | 35/173 (20.2) | 24/185 (13.0) | 0.064 | 16/63 (25.4) | 10/38 (26.3) | 0.918 |
| NEC ≥stage Ⅱ | 27/174 (15.5) | 23/185 (12.4) | 0.399 | 12/63 (19.1) | 6/38 (15.8) | 0.679 |
| ROP, operation | 48/142 (33.8) | 85/165 (51.5) | 0.002* | 28/54 (51.9) | 12/31 (38.7) | 0.243 |
| ROP ≥stage III | 71/173 (41.0) | 97/185 (52.4) | 0.031* | 32/63 (50.8) | 20/38 (52.6) | 0.858 |
| Need for home oxygen  at discharge | 17/144 (11.8) | 17/171 (9.9) | 0.595 | 10/30 (33.3) | 4/27 (14.8) | 0.105 |
| Length of hospital days | 132.0 (40.0) | 128.6 (34.1) | 0.304 | 200.9 (79.5) | 185.5 (81.9) | 0.218 |
